# Pig Genome Editing for Agriculture: Achievements and Challenges

**DOI:** 10.3390/ijms262412140

**Published:** 2025-12-17

**Authors:** Elena Mikhaylova, Emil Khusnutdinov, Mikhail Terekhov, Daniil Pozdeev, Oleg Gusev

**Affiliations:** 1 Institute of Biochemistry and Genetics, Ufa Federal Research Centre, Russian Academy of Sciences, 450054 Ufa, Russia; 2 Life Improvement by Future Technologies (LIFT) Center, 121205 Moscow, Russia; 3 Intractable Disease Research Center, Graduate School of Medicine, Juntendo University, Tokyo 113-8421, Japan

**Keywords:** CRISPR/Cas, ZFN, TALEN, knock-in, knockout, virus resistance, meat quality, meat productivity, pig genomics, SCNT, RNP

## Abstract

The remaining problems in pig farming may no longer be solved with traditional methods. The search for genetic variants associated with desired characteristics and involvement of animals with superior genetics in breeding programs is rarely effective for polygenic traits and pleiotropic genes. The lack of diversity in the germplasm also limits the use of breeding, but some beneficial mutations that did not occur naturally can be introduced manually via genome editing methods. Mutations discovered in other species, such as cattle, can be reproduced in pigs. Traits that were previously pursued for centuries might be achieved by genome editing in a few years. Enormous progress has been made in producing pigs resistant to viruses and in increasing meat productivity and quality. But there are still pressing problems such as lameness and damaging behaviors that probably cannot be solved without genome editing techniques. Their wider application is complicated by the requirement for large amounts of biomaterial, surgical manipulations and cell culture, as well as by the shift towards biomedical research. This review concentrates on the main achievements and challenges in pig agricultural genetics that can be addressed by genome editing.

## 1. Introduction

The global food crisis requires the improvement of agriculture efficiency to provide people with high quality nutrition and solve the problem of hunger. The rising population increases the demand for protein. Pork is the most affordable meat, accounting for 30% of the world’s total meat production, of which more than 50% originates from Asia and around 20% from Europe. The US is the world’s second-largest pork producer after China [1,2,3]. Pigs have high feed efficiency, large litters, and a short lifecycle, which make them indispensable for human welfare. But, the rapidly developing society poses new challenges to pig farming. Emerging agricultural problems, such as outbreak and spread of infectious diseases, new health issues caused by intense selection for meat productivity, and stress-induced behavior, require a transformative shift in global pig production [4,5]. It is impossible to achieve sustainable and efficient solutions to modern pork productivity and quality challenges without genetics.

*Sus scrofa* genome is packaged within 18 autosomes plus X and Y sex chromosomes, has around 21,000 protein-coding genes, and shows high synteny with the human genome. Since it was first sequenced and annotated in 2012, pig farming has entered the post-genomic era [6]. Genetic markers have become widely used to assess animal productivity and guide selective breeding. Genome-wide association studies (GWAS) which link genetic variants to economically important traits have made it possible to identify genes and genetic variants responsible for a range of specific traits, increase the rate of genetic improvement, and reduce inbreeding [7,8]. Currently, more than 350 complete genomes of 48 various pig breeds and wild boars are available. Analyses revealed that in China and Europe pigs were domesticated independently, and there are many domestication-driven genetic signatures in pig breeds. Sequencing of full pig genomes has accelerated the development of genome editing and cell technologies, enabling precise targeting and innovative applications in agriculture and biomedicine [9].

Pigs are unusual among mammals. They have very few, poorly functional sweat glands and low amounts of brown adipose tissue, which is essential for maintaining body temperature. Temperature sensitivity in pigs leads to significant economic losses due to the differences in the optimal environmental temperature for piglets and adult pigs [10]. Genetic studies demonstrate that heat sensitivity in pigs developed due to the loss of functional UCP1 (uncoupling protein 1) about 20 million years ago [11]. Nevertheless, pig organs, as well as immune response, are highly comparable with those of humans, making xenotransplantation and establishment of disease models possible [12]. This is why, despite many unsolved problems in pig farming, biomedical research on pigs is currently being more intensively conducted and is more visible than agricultural genetics. Agricultural research is usually industry driven, and the results become property of the breeding companies, which slows global progress in pig farming and creates food security risks. The consolidation of pig breeding within a few global companies contributes to a reduction in genetic diversity and loss of local adaptations [13,14]. Therefore, independent research in the field of agricultural pig genetics is now more important than ever.

Modern pig industry uses a crossbreeding system built around a maternal F1 from white breeds and a colored terminal boar, which maintains genetic purity and superior traits within breeds, and then combines breeds to exploit heterosis [15]. Commercial pork production relies on a few standard cross systems, involving Landrace, Yorkshire, Duroc, and Large White breeds; however, in Europe alone there are more than 42 endangered indigenous breeds, and 48 are considered extinct [13]. Even the standard breeds such as Landrace undergo a large number of mergers and lose the genetic diversity that was present in the discontinued lines [14].

The integration of new technologies such as genome editing into swine breeding offers transformative potential for enhancing the competitiveness of local pig breeds and restoring agricultural sustainability. Traits that previously took years to study and breed can now be achieved in just a few generations [16]. Genome manipulations also offer a unique opportunity to induce mutations that are absent in natural populations and may provide undeniable advantages to indigenous breeds in a very short period of time.

Unlike traditional approaches, CRISPR/Cas is a tool mainly used by research institutes and universities. Sharing knowledge in this area will accelerate progress in understanding complex traits and help overcome biological barriers faced by traditional methods, such as co-selection of unwanted features.

This review summarizes the latest achievements and challenges in the discovery of genetic patterns influencing important traits in pigs, with an emphasis on the potential for genome editing applications.

## 2. Main Achievements and Challenges in Pig Agricultural Genetics

Since the introduction of genetic markers and genomic tools such as GWAS, sequencing and transgenesis, significant progress has been made in pig production, allowing breeders to select superior animals at a young age based on their genetic profile [17]. The single genes affecting growth rate, meat quality, disease resistance, and reproductive efficiency were discovered [18]. Later high-density SNP (Single Nucleotide Polymorphism) arrays built on thousands of DNA markers have become standard tools in genetic research and breeding. The 60K chip and its successors have enabled the development of the most productive lines, which have become popular among pork producers around the world [19]. Polymorphisms closely associated with beneficial effects have been successfully used in breeding, but many important agricultural traits are more complex and may require genetic variants that do not exist in natural populations.

### 2.1. Meat Quality and Productivity

Favorable alleles can be selected for gene encoding the fatty acid-binding protein (H-FABP) which controls fat thickness; the *estrogen receptor* (*ESR*) gene associated with multiple pregnancy; the gene encoding the skeletal muscle ryanodine receptor (RYR1) which provides resistance to stress; the *insulin-like growth factor-2* (*IGF-2*) gene which affects the growth and development of muscle; *calpastatin* (*CAST*) gene associated with meat tenderness; and 5′-AMP-activated *protein kinase subunit gamma-3* (*PRKAG3*) gene which affects meat pH and color. For example, the 1843C>T polymorphism (p.Arg615Cys) in the *RYR1* gene causes the development of malignant hyperthermia, also known as Porcine Stress Syndrome, characterized by uncontrolled release of calcium into the muscle cells under stress conditions resulting in rapid heat production, increased heart rate, and respiratory distress. The same polymorphism is associated with an increase in carcass leanness, but the introduction of genetic testing has reduced the frequency of the mutant allele in pig populations [20].

GWAS analysis has allowed researchers to identify the promising candidate genes associated with meat quality, estimated by residual glycogen, glucose, and lactate in the longissimus muscle: *transmembrane and coiled-coil domains 1* (*TMCO1*), *LRAT Domain Containing 1* (*LRATD1*), and *creatine kinase B* (*CKB*) [21]. Gene silencing by RNA interference can help to determine the role of the candidate genes discovered by GWAS, transcriptome analysis and whole-genome sequencing. For example, siRNA interference experiments with porcine subcutaneous and intramuscular adipocytes revealed that Galectin-12 (LGALS12) is an important regulator of fat deposition in pigs [22], but application of this technology is quite rare.

### 2.2. Hereditary Diseases

It was shown that anal atresia in pigs is associated with two major mutations in the zinc finger transcription factor gene *GLI2*, which is involved in the embryonic development of the nervous system and skeletal system of the body [23]. A deletion in the *spectrin beta non-erythrocytic 4 protein* (*SPTBN4*) gene affects localization of ion channels in myelinated nerves and causes severe myopathy and postnatal mortality [24]. Congenital splay leg syndrome in piglets causes lateral abduction of the hind limbs and inability to walk. The condition is associated with the F-box only protein 32 (FBXO32) which is highly expressed during muscle atrophy, multifunctional Neuronal protein 3.1 (P311) responsible for cell differentiation, and homer scaffold protein 1 (HOMER1) involved in skeletal muscle differentiation [25].

### 2.3. Infectious Diseases

DNA tests can currently detect susceptibility to several infectious diseases: polymorphism in the *FUT1* gene and the *mucin 4* (*MUC4*) gene determine the susceptibility to *Escherichia coli*, and the *natural resistance-associated macrophage protein 1* (*NRAMP1*) gene is involved in resistance to *Salmonella* [26].

The search for receptor genes of various viruses is of great importance to obtain resistant breeds. Porcine reproductive and respiratory syndrome virus (PRRS) is a highly contagious and economically important pathogen for the swine industry worldwide. It causes respiratory disease with a 12–15% mortality rate in young pigs and reproductive failure in pregnant sows, including late-term abortion and low-birth-weight piglets [27]. Naturally occurring PRRS-resistant pigs do not exist. Molecular receptors such as heparan sulfate, vimentin, CD151, CD163, CD169, and CD209 have been confirmed to be essential for PRRSV infection [28]. SNPs in the *CD163*, *CD169*, and *RGS16* receptor genes have been associated with resistance to PRRSV as well as Porcine Circovirus Type 2 (PCV) [29]. Polymorphisms in genes of guanylate-binding proteins involved in innate immune responses to bacterial and viral infections (GBP1 and GBP5) also alter the animal’s resistance to PRRSV, probably by increasing production of antiviral cytokine (IFN-α) and T cell activation [30,31]. Among them, *CD163* is considered to be the major PRRSV receptor and determines cellular sensitivity to the virus [32].

Foot-and-mouth disease virus (FMDV) causes myocarditis in young pigs and severe lameness in adults due to the blisters and erosions on the feet. Inactivated vaccines can be used, but they must match the circulating serotype. Complete resistance can be achieved by transgenesis. All transgenic pigs expressing FMDV-specific short interfering RNA were asymptomatic after intramuscular injection with 100 LD50 of FMDV, demonstrating the huge potential of this technology [33].

Alphacoronaviruses such as Transmissible gastroenteritis (TGEV), Porcine Epidemic Diarrhea (PEDV), and porcine deltacoronavirus (PDCoV) are the major causes of neonatal diarrhea in pigs, resulting in high mortality rates. Moreover, coronaviruses frequently cross species barriers and can infect humans. A variety of vaccines with variable efficiency have been developed for pigs, mostly for oral vaccination of sows [34]. Aminopeptidase N (ANPEP, or CD13) is considered an entry mediator for pig coronaviruses, and 4 integrins (αvβ1, αvβ3, αvβ6, and αvβ8) and heparan sulfate are known receptors of the virus; no single major-effect resistance gene has been validated for these infections [35].

Classical swine fever (CSFV) is a contagious virus with varying strain-dependent severity and clinical signs. It has been shown that CD46 is its main receptor, and heparan sulfates serve as additional cellular factor [36]. It was suggested that viral protein interacts with porcine Poly (rC)-binding protein 1 (PCBP1) to enhance replication [37]. It was also discovered that the host molecular chaperone DnaJ homolog subfamily C member 14 (DNAJC14) acts as a core component in the replication of pestiviruses, including CSFV and bovine viral diarrhea virus (BVDV) [38].

Senecavirus A (SVA) has the ability to mimic the signs of more lethal diseases such as FMDV, vesicular stomatitis, vesicular disease, and vesicular exanthema, causing blisters, lameness, and temporary reproductive problems. There is currently no vaccine to treat it [39]. It has been demonstrated that anthrax toxin receptor 1 (ANTXR1) is a cellular receptor for SVA [40].

The swine, human, and avian influenza viruses pose a significant threat to pig herds, causing acute respiratory disease with fever, coughing, lethargy, and loss of appetite. This disease plays a critical role in human pandemic risk as pigs act as “mixing vessels” for influenza viruses, resulting in the emergence of new pathogenic variants, such as 2009 pandemic H1N1 strain. Vaccines exist but offer poor cross-protection. It is known that the transmembrane serine protease 2 (TMPRSS2) influences viral pathogenicity and infectivity [41,42]. Interferon-induced GTP-binding protein Mx1 serves as a downstream effector of type I interferons and confers resistance to a broad spectrum of viruses. Transgenic pig cells overexpressing Mx1 protein demonstrated increased resistance to influenza A virus and CSFV [34]. However, early experiments aimed at the generation of resistant pigs by transformation with murine *Mx1* gene revealed that it might be deleterious to the organism [43].

Pseudorabies virus (PRV) is a pathogen from family Herpesviridae that causes Aujeszky’s disease, characterized by wide range of clinical signs, high mortality rate in piglets, and reproductive failure in sows. In adult pigs virus can exist in a latent state and reactivate during periods of stress. Vaccination facilitated PRV eradication in domestic swine in different countries, including the EU and US. Immunoglobulin-related transmembrane cell–cell adhesion molecules Nectin 1 and 2 are considered PRV primary receptors, and heparan sulfate serves as co-receptor. Herpesvirus entry mediator and poliovirus receptor are involved in infection with other herpesviruses, but their role in the development of Aujeszky’s disease has not been confirmed [44].

African swine fever virus (ASFV) causes a hemorrhagic disease with 90–100% mortality rate. Its environmental stability, long incubation period, and genetic complexity make disease control extremely challenging. The ASFV binds the RELA subunit of the NF-κB transcription factor which plays a key role in regulating immune response upon infection to suppress the host’s immune response [45]. Warthogs, which are asymptomatic and therefore act as virus reservoir, differ from domestic pigs by three amino acids in the RELA protein [46], but it is unlikely that specific RELA variants can confer ASFV resistance, because the virus uses multiple entry paths. Porcine CD1d protein appears to be another binding partner for ASFV virions, which mediates its entry into the host cells. Yet its knockdown only inhibited the infection and did not ensure full resistance [47]. At the moment, there is no safe and effective vaccine or treatment for this disease.

Japanese encephalitis virus (JEV) is a mosquito-borne flavivirus that primarily affects pigs as amplifying hosts. They rarely show severe clinical disease, but the virus can cause encephalitis and reproductive failures. Pigs develop high viremia levels, attracting and infecting mosquitoes, which then transmit the virus to humans. Until recently, the receptors and attachment factors for this virus were unknown, possibly due to its geographical distribution being limited to Asia [48,49].

There are also several other viruses that have not received significant attention due to their asymptomatic nature, such as Sapelovirus, Kobuvirus, and Bocavirus [50,51,52]. Such viruses are not considered dangerous; however, they can participate in co-infection or may pose risks in the future. Most of the significant swine viral diseases are well studied, and the limited number of target genes required for breeding of resistant pigs or genetic modifications are identified.

### 2.4. Polygenic Diseases

Despite the significant advances achieved in pig breeding through selection for single genes, sophisticated markets and growing population force pork producers to constantly improve pig genetics. But, many important agricultural traits are polygenic, while for others the genetic mechanism of formation is still unknown. Their discovery requires genotyping of many animals with contrasting phenotype and big data analysis. This can be problematic for traits that are hard to diagnose or detect, such as lameness and damaging behaviors.

Lameness (or leg weakness) is a common problem with a variety of clinical signs that are likely caused by several factors that are difficult to distinguish from each other. Decreased mobility, limping, abnormal gait, swollen joints, and hoof lesions that are often diagnosed as lameness can be the result of not only genetics but also nutritional and management issues, as well as bacterial infections [53]. A lot of genes have been associated with this condition. Nan et al. proposed two candidate quantitative trait loci (QTL) and three potential candidate genes associated with bone mineral density. Cannabinoid receptor 2 (CNR2) has a significant role in regulating bone metabolism in humans and mice. Lin-28 homolog A (Lin28a) can enhance repair in adult tissues, such as ears, following injuries [54]. Zinc finger and BTB domain containing 40 (ZBTB40) was characterized as a regulator of osteoblast activity and bone mass in mouse models [55]. When osteochondrosis scores were used for association analysis, the metalloproteinase 3 (MMP3) involved in extracellular matrix degradation in joints and transforming growth factor beta 1 (TGFβ1) appeared to have an effect on this leg-weakness-related trait. The *TGFβ1* gene, known to be deficient in chondrocytes at sites of osteochondrosis, stimulates their proliferation [56]. Bone morphogenetic protein receptor 1B (BMPR-IB) is also a key regulator of chondrogenesis and osteogenesis [57]. Polymorphisms in *Fas-associated factor 1* (*FAF1*) gene which enhances apoptosis, and *parathyroid hormone type I receptor* (*PTH1R*) gene which regulates cartilage growth and chondrocytic apoptosis, were associated with osteochondrosis lesions in pigs. G allele of the *keratin 8* (*KRT8*) was strongly associated with better bone mineralization; however, the impact of this gene on the development of function of leg weakness pathology is unclear [58]. Breeding pigs with a reduced risk of lameness is complicated by the involvement of the same genes in formation of other traits such as productivity and meat quality. For example, positive selection for the reduced fat content resulted in the maintenance of c.820G>T; p.E274* mutation in the *myostatin* gene (*MSTN*), which is a dominant inhibitor of skeletal muscle development. However, in the homozygous state this mutation causes lameness in piglets [59].

Damaging behaviors include biting of tails and ears, which can lead to infection, stress, lameness, decreased growth rate, meat quality, reproductive performance, and even death of the victims. Aggressive pigs consume more feed and grow faster, so negative selection of calm animals could take place. GWAS analysis revealed that specific polymorphisms in neurotransmitter genes *dopamine receptor* gene (*DRD2*), *serotonin transporter* (*SLC6A4*) which regulates the availability of serotonin, *monoamine oxidase A* (*MAOA*), and *RYR1* gene are associated with porcine aggression. DRD2 affects the rate of apoptosis in porcine neuroglial cells by binding negative regulatory factor interferon 2. MAOA contributes to psychiatric disorders in humans by catalyzing oxidation of serotonin and dopamine [60].

### 2.5. Boar Taint

One of the problems in pig farming is the excess number of boars, which usually make up half of the litter. Only a small number of males are required for reproduction, and the others get castrated to prevent a boar taint, which is an unpleasant urine- or fecal-like odor and flavor in the meat caused by a sex pheromone androstenone, and aggression. Intact boars convert feed into muscle more efficiently and have leaner carcasses, but their meat is unacceptable to consumers due to unpleasant smell [61]. Immunization against gonadotrophin releasing factor (GnRF) can be used shortly before slaughter to suppress testicular function as an alternative to castration [62]. Sexing of sperm in pigs is problematic due to its susceptibility to stress caused by sorting and high dilution rates which are insufficient for optimum fertility in the female [63]. GWAS analysis revealed candidate genes associated with androstenone content such as *cytochrome P450* genes cluster, *sulfotransferases SULT2A1* and *SULT2B1*, the *β*-chain of the *luteinizing hormone* (*LHB*) gene, *3β-hydroxysteroid dehydrogenase* (*3βHSD*), and *17β-hydroxysteroid dehydrogenase* (*17βHSD*) [64,65].

### 2.6. Introduction of Foreign Genes

Studying native genes can only improve existing qualities, and the acquisition of fundamentally new traits is only possible with the help of genetic engineering and genome editing.

For example, there are approaches to increase meat quality by transgenesis. Expression of spinach-derived Δ12 fatty acid desaturase increased linoleic acid content in white adipose tissue of transgenic pigs [66]. Expression of an n-3 fatty acid desaturase from *Caenorhabditis elegans* enriched pork meat with omega-3 fatty acids [67].

Pigs lack the extensive microbial fermentation chamber to break down complex fibers and do not produce enzymes like phytase or cellulase. Ineffective digestion in pigs leads to the waste of significant amounts of phosphorus and energy from fodder, reduced growth, and release of excess nutrients into the environment. The most widely practiced strategy is to introduce feed additives such as exogenous enzymes, probiotics, and prebiotics. An alternative strategy to enhance feed digestion is to produce genetically engineered animals that express the missing enzymes. Transgenic pigs expressing salivary phytase and those expressing three microbial enzymes, β-glucanase, xylanase, and phytase, had reduced fecal phosphorus levels and did not require phosphate supplementation [68,69,70]. Pigs also successfully expressed fungal cellulase which was active in their pancreas [71] and bacterial β-Glucanase [72]. Multi-transgenic Pigs expressing pectinase, xylanase, phytase, cellulase, and β-glucanase were generated in 2020, but the pectinase expression level was lower than expected [73].

Expression of the bovine and human *alpha-lactalbumin* genes improved the productivity of transgenic sows and accelerated the growth of their piglets [74,75]. Transgenic cloned pigs that express an antimicrobial human lysozyme (hLZ) in the milk were produced to inhibit growth of K88 *E. coli* in their piglets. Animals that consumed the milk recovered more quickly from bacterial-induced diarrhea [76]. To protect pigs from aflatoxin that can be present in the fodder, they were transformed with bacterial *aflatoxin-detoxifizyme* (*ADTZ*) gene which increased their tolerance to aflatoxin [77].

Transgenic pigs expressing small interfering RNAs (siRNAs) against several viruses including PERV, FMDV, and PRRS were produced [78,79,80].

Overexpression or downregulation of endogenous genes in transgenic pigs also can benefit some agricultural traits. Higher feed efficiency and meat production was achieved in transgenic pigs with doxycycline-inducable pig growth hormone expression [81]. Thermotolerance in pig primary fibroblasts was improved by overexpression of heat shock protein 70 (HSP70) [82]. Inactivation of the porcine *α-1*, *3-galactosyl-transferase* (*GGTA1*) gene prevents allergic reactions in people with alpha-gal syndrome [83]. GalSafe™ pigs lacking alpha-gal epitope due to the insertion of a polyA cassette into 9th exon of the *GGTA1* gene were approved by US Food and Drug Administration (FDA) in 2020 [83,84]. So far, this is the only case of approval of GM pigs for human consumption. Another project called EnviroPig, developed at the University of Guelph in Ontario and approved in Canada in 2012, had been suspended due to the complexities of regulatory frameworks and public concerns. These transgenic pigs were characterized by a reduction in phosphorus pollution by up to 70% due to the production of phytase [85].

Thus, many problems in pig farming can be solved through genomic selection, transgenesis, or genome editing. The latter have the advantage of faster achievement of the desired result and allow for the production of animals with traits that are naturally absent in pigs. Biotechnology also gives opportunity to study the mechanisms of development of certain diseases and the formation of economically valuable traits, determining the contribution of individual genes in those considered polygenic. Manipulations of pig genomes are much less efficient than in mice and humans, probably due to the difficulty of maintaining commercial pig breeds in laboratory conditions and the regulatory restrictions in most countries. Nevertheless, pigs are better studied than other livestock due to their potential for use in biomedical research.

## 3. Principles of Pig Genome Manipulations

**Figure 1:**
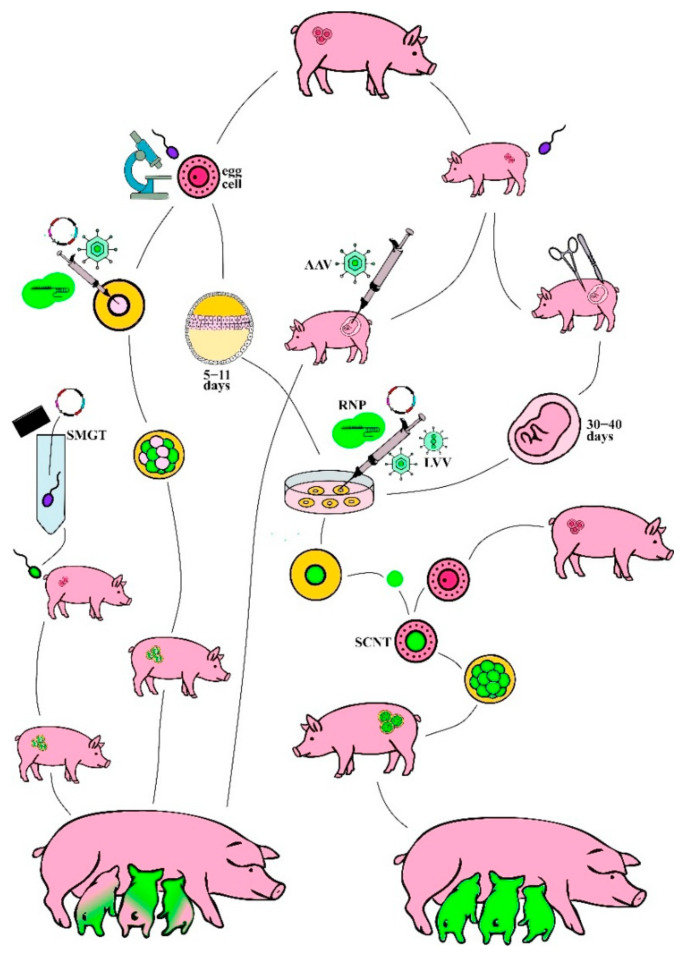
Delivery methods for genetic engineering and genome editing tools. The two main branches demonstrate manipulations with egg cells (left) and naturally produced embryos (right) as well as sperm-mediated gene transfer (SMGT) when male cells get treated with plasmids. Egg cells fertilized in vitro can be either used for direct delivery of ribonucleoprotein (RNP), plasmid, or adeno-associated virus (AAV) vector, as well as for the establishment of a cell culture. Cell cultures can be transformed with plasmids, RNPs, AAV, or lentiviral vector (LVV), and stable edited lines are further used for somatic cell nuclear transfer (SCNT). Only AAV and sperm-mediated gene transfer (SMGT) were employed in pigs in vivo. Depending on the chosen approach, the progeny can be either chimeric (left) or homozygous (right).

Pig was one of the first transgenic animals, created in 1985 by pronuclear microinjection into fertilized zygotes [86]. Since then, a broad toolbox of methods was developed, each with benefits and limitations (Figure 1). Pig genome manipulations include genetic modifications with foreign DNA and genome editing, which allow introducing precise point mutations that are indistinguishable from natural ones. Both approaches primarily rely on a suite of complementary technologies, each with distinct strategic advantages. The genome manipulations can be conducted either on in vitro fertilized egg cells or naturally produced embryos. To achieve homozygous progeny in the first generation, somatic cell nuclear transfer (SCNT) and the establishment of a cell culture are required. In vivo approaches and transformation of embryos are more likely to result in chimeric progeny, carrying various somatic mutations. Traditional genetic engineering has serious drawbacks, including random integration and the risk of integration of multiple copies of genetic construct, potentially disrupting functional genes or regulatory elements. In most countries, genetically modified animals are banned and therefore can only be used for research purposes and in medicine. It is more likely that genome-edited pigs have more prospects for application in agriculture due to easier safety assessment and regulation; however, transgenic pigs might be soon employed for organ transplantation and other treatments that can save human lives.

Genome-editing tools include mega-nucleases, zinc finger nucleases (ZFNs), transcription activator-like effector nucleases (TALENs), and the CRISPR/Cas system. All of these approaches employ nucleases that can induce double-strand break (DSB) in a specific site of the genome, which is repaired by the cellular mechanisms. Reparation can occur either by non-homologous end joining (NHEJ) which can cause mutations (knockouts), or homology-directed repair (HDR) which allows insertion of new DNA from a donor sequence (knock-ins) [87,88].

ZFN technology is characterized by high cost and complexity due to the requirement for assembly of proteins that recognize DNA triplets. TALENs have very low off-target effects and allow flexible targeting but require designing and assembling large proteins for each target. The CRISPR/Cas system employs a ribonucleoprotein (RNP) consisting of Cas and guide RNAs (gRNAs), which are designed to target a specific site of the genome. The flexibility of the system is restricted by a PAM sequence which must be adjacent to the gRNA.

ZFN and TALEN pioneered genome editing but have been largely eclipsed by CRISPR/Cas, which is the most promising method due to its unprecedented precision, versatility, efficiency, and accessibility. The diversity of Cas proteins and their modifications allow for base and prime editing, transcriptional regulation, and even RNA editing [89]. Most of these approaches have not yet been used in livestock, but there is no doubt that the future of genetic improvement in pigs lies with CRISPR/Cas. Unlike genetic engineering, this technology is considered precise and safe in many countries, if foreign DNA is not involved. Therefore, generation of animals with edited genomes is the final goal.

Embryos are the most common objects of manipulation that can be subjected to pronuclear microinjection or electroporation and implanted to recipient females. Compared to microinjection, electroporation inflicts less physical damage on the zygotes. Viral particles can be directly injected into the oviduct of a pregnant animal shortly after fertilization [90]. Sperm-mediated gene transfer (SMGT) was also successful in generating transgenic pigs when sperm was cocultivated with plasmid DNA and lentivirus vector and used for artificial insemination [91,92,93].

Mosaicism and chimerism are the main disadvantages of these methods, due to the late embryonic genome activation in pigs. Their genome is transcriptionally silent until the 4-cell stage, compared to 2-cell stage in mice. Mature oocytes are also transcriptionally inactive [94,95]. Integration of foreign DNA and DSB repair depend on host DNA repair pathways which are not functional in early-stage embryos. Delivered DNA or RNPs are processed unequally across blastomeres and can degrade too early, resulting in 20–70% mosaicism with multiple modifications [88]. Several breeding steps are required for generation of homozygous animals. Given the 4 months gestation periods and 6–9 months to reach reproductive age, breeding of hemizygous and especially mosaic pigs to homozygosity can take years [35]. The primary strategies to reduce mosaicism in pig genome editing focus on ensuring the editing event occurs before the embryo’s first cell division. This can be achieved by the delivery of a ready-to-cut RNP complex instead of DNA, which allows avoiding transcription and translation steps. However, it has not yet been proposed how to influence the initiation of DNA repair mechanisms.

Establishment of a stable fetal fibroblast cell line with desired mutations, followed by somatic cell nuclear transfer (SCNT) from somatic cells to oocytes, deals with the problem of mosaicism but brings new complications. Impaired embryonic development after SCNT contributes to high rates of embryonic and fetal loss. Embryos suffer from incomplete epigenetic reprogramming of donor cell nuclei, including DNA methylation, histone acetylation and methylation, miRNA regulation reprogramming, and downregulation of X chromosome-linked genes, resulting in the wrong chromatin structure, epigenetic marks, and transcriptional profile. This may lead to a longer gestation and oversized offspring with organ malfunctions and immunological deficiencies [96,97,98,99].

Several methods to ensure normal development of the embryos after SCNT have been proposed. Activation of pluripotency genes and cell reprogramming with hypomethylating agents, maternally derived histones, and histone deacetylases or specific transcription factors can be achieved by various treatments, including genetic engineering [96,100,101,102,103,104,105,106,107,108]. Pluripotent stem cells (PSCs) obtained by reprogramming somatic cells are called induced PSCs (iPSCs) [109,110,111,112]. Although reprogramming is often incomplete, and expression level of endogenous pluripotency genes in iPSCs remain unstable.

Another approach to improving SCNT is the use of embryonic stem cells (ESCs) derived from preimplantation porcine blastocysts and more stable and versatile expanded potential stem cells (EPSCs) with broad developmental capacity [113,114,115,116]. Maintaining pluripotency requires complex cell culture systems [114,115,117].

However, fetuses at ~30–40 days of gestation are used for establishment of a fetal fibroblast cell culture, and E5-E11 inner cell mass or epiblasts at embryonic disc stage are required for ESCs and EPSCs. EPSCs express key pluripotency genes but also maintain genetic stability [113]. Unlike ESCs, fetal fibroblasts are characterized by slow proliferation and low recombination rates. They are also easy to isolate and culture [35]. All cell cultures are most easily transformed via electroporation, but lipofection and viral vectors can also be used.

Therefore, direct transformation of pig embryos as well as cloning via SCNT and in vitro embryo culture are well-established, enabling easy use of CRISPR, TALEN, or ZFN genome editing and genetic transformation. In vivo approaches remain the least developed, but in recent years they have attracted attention due to their potential for use in industrial settings. The choice between cell types and delivery method depends on the specific requirements of the project, such as the complexity of genetic modifications, regulatory considerations, and available resources.

## 4. Delivery Platforms for Molecular Tools

**Figure 2:**
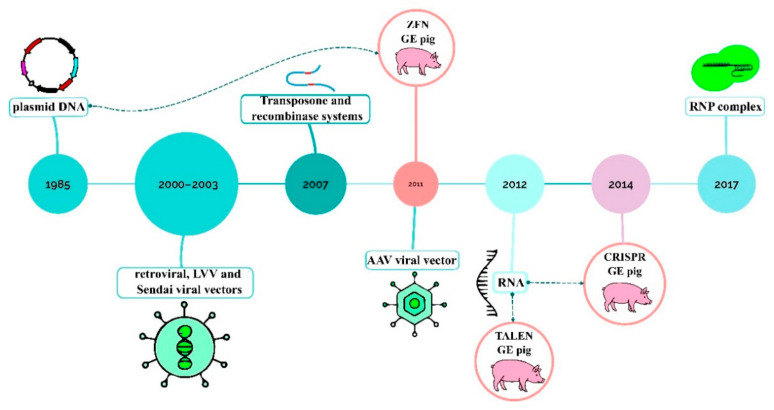
Timeline of molecular tool delivery platforms application in swine, highlighting the emergence of the first genome-edited (GE) pigs.

Delivery platforms are crucial for introducing transgenes or genome editing tools into pigs, and their evolution has been the primary driver of progress in the field. They include linear and plasmid DNA, recombinase and transposon systems, RNA, viral vectors, and RNP complexes (Figure 2).

### 4.1. Delivery Platforms Initially Designed for Genetic Engineering

The first transgenic pigs were created using linearized plasmid DNA [86]. Linearized DNA integrates with greater efficiency than supercoiled DNA, but the transformation rate is ≈1–4%, which is 10-fold lower than in mice.

In the next decade SCNT became the most widespread method. Plasmid microinjection had very low success rates in fetal fibroblast cultures therefore viral vectors were developed to increase integration efficiency. In 2001, a vector based on Moloney murine leukemia virus was used to transform porcine oocytes with green fluorescent protein (GFP) gene, resulting in one transgenic pig in a litter [118]. In 2003, HIV-1-derived lentiviral vector (LVV) carrying *GFP* gene was propagated in human 293T cells and delivered to porcine embryos with 70% efficiency. The transgene was successfully transmitted through the germline [119]. In 2004, an equine infectious anemia virus (EIAV)-derived lentiviral vector was used to deliver GFP transgene into porcine embryos and egg cells with 31% efficiency [120]. Sendai RNA virus that replicates entirely in the cytoplasm of infected cells has also been applied in pigs. Its genetic material is expressed transiently without integration into the host genome [121,122]. Delivery rate of transgenes via inactivated Sendai virus to porcine cells reaches 30% [123].

Human adeno-associated viruses (AAV) are frequently used in pig research. In 2011, hybrid AAV vector assembled from serotypes 2, 8, and 9 was applied to generate genetically modified pig models for human diseases through SCNT [124]. AAV9 is especially interesting due to its ability to cross the blood–brain barrier, broad tissue tropism, and systemic spread. AAV6 proved the most efficient in embryos [90]. Viral vectors may pose some biosafety concerns. For example, they can trigger immune responses such as inflammation or integrate into the genome near oncogenes or regulatory elements. There is also a risk of recombination with endemic swine viruses which can have epidemiological consequences in industrial conditions. Nevertheless, non-integrating, non-replicating AAV vectors are considered non-pathogenic and pose minimal risk when manufactured under proper conditions [125].

In 2007, four transposon systems—Sleeping Beauty, Tol2, piggyBac, and Passport with Cre and Flp recombinases included in the constructs—were tested in porcine cells. Recombinases can excise or invert sequences located between engineered target sites. This approach made it possible to deliver up to 15 transgenes per cell [89]. The piggyBac system became widely used in pigs. It includes transposase and a gene of interest which is cloned between inverted terminal repeats. Transposase recognizes these repeats, excises the donor fragment, and integrates it into the host genome randomly at TTAA sites. This approach contributed to a 30-fold increase in target gene integration frequency in fibroblasts. Direct injection of a piggyBac plasmid to one-cell embryos yielded transgenic piglets at ~8% rate [126,127,128,129]. Efficiency of Cre-loxP recombination in transgenic zygotes can reach 81.9% [130].

### 4.2. The Era of Genome Editing

The first genome-edited pigs were created in 2011 using ZFNs. Porcine *GGTA1* gene and *GFP* were successfully knocked out in transgenic pigs. Primary porcine fetal fibroblasts were electroporated with ZFN plasmid followed by SCNT. The efficiency of the ZFNs was from 1 to 5% [131,132]. In 2012 knockout of the *LDL receptor* (*LDLR*) gene was performed via TALEN to produce swine models of familial hypercholesterolemia disorder. Injection of TALEN mRNA into zygotes efficiently induced gene knockout as well as TALEN plasmid in fetal fibroblast culture [133].

The CRISPR/Cas approach was first used in pigs to knock out the *vWF* gene responsible for von Willebrand disease in 2014. One-step editing through direct zygote injection of Cas9 mRNA and sgRNA resulted in generation of monoallelic and biallelic mutant pigs that could serve as a disease model [134]. In the same year, CRISPR/Cas9 editing of the e*GFP*, *CD163*, and *CD1D* genes in pigs was successfully conducted using transformation of fibroblasts with a plasmid followed by SCNT, and in vitro delivery of mRNA coding for Cas9 and gRNAs into porcine zygotes [135]. Generation of gene-edited miniature pigs via injection of zygote with Cas9 mRNA and sgRNA was 100% efficient [136,137]. The *TMPRSS2* gene, which provides resistance to certain types of influenza viruses, was edited with 92–100% efficiency [137].

Thus, since the integration of foreign DNA is undesirable in genome editing, a new delivery method via RNA encoding *Cas* gene and separate gRNAs has been developed. It is noteworthy that genome editing has been implemented primarily in biomedical research.

The initial CRISPR-induced knock-in in pigs was documented in 2015 [138]. A 9 kb DNA fragment including *GFP* gene was successfully introduced to a pH11 “safe harbor” locus in porcine fibroblasts using two plasmids and a linearized DNA donor. The efficiency was as high as 54% with drug selection and 6% without drug selection. However, out of 243 embryos generated by SCNT, only one piglet was born.

In 2017 RNP complex was used for the first time to target the downstream region of *collagen* (*COL1A*) gene by direct injection of Cas9 ribonucleoprotein complex and donor DNA sequences into porcine zygotes. Like RNA, RNP complexes are a unique delivery method for genome editing. This approach allowed the introduction of pseudo attP sites in the target region to enable phiC31 integrase-mediated introduction of transgenes [139]. Lipofection of RNPs to 1- to 8-cell stage embryos produced *MSTN*-mutant pigs with 77% efficiency [140]. RNPs can also edit somatic cells by electroporation/lipofection. A multi-gene RNP system allowed the editing of four loci in porcine fetal fibroblasts simultaneously [141].

LVV was first used in 2016 to create porcine primary fibroblasts carrying the tetracycline-inducible *Cas9* gene [142]. However, this method is rarely used in genome editing because the resulting animals are transgenic.

The use of AAV for genome editing only began in 2023. Intravenous delivery of CRISPR/Cas9 in AAV vector has been used to edit the genome in animal brains in vivo to treat Huntington’s disease. Editing also took place in germline cells [143]. Recent papers demonstrate the possibility of genome editing through oviductal injection of AAV vectors into pre-implantation pig embryos, but the efficiency was just 3.8% [90]. This is a promising tool, enabling in vivo genome editing, which is especially important when animal housing in laboratory conditions is not possible. Generation of genome-edited piglets require numerous sows and facilities to house them. In vivo experiments can be carried out directly on the farm without significant financial costs, but AAV has limited capacity (~4.7 kb). Large *Cas9* (~4.2 kb) + promoter + sgRNA + regulatory elements often push that limit.

### 4.3. New Genome Editing Technologies

CRISPR/Cas can be used not only for precise modification of known targets, but also to identify new genes involved in specific biological processes or genes responsible for advantageous traits. Cell cultures are infected with a CRISPR/Cas screening library with multiple gRNAs, creating a population of cells where each cell has a different gene knocked out. These cells are then exposed to a stress factor, and the surviving cells are analyzed for the gene knockout. In 2020 the first porcine genome-scale CRISPR/Cas9 knockout library with ~85,000 sgRNA constructs was used in porcine kidney cells to identify host factors essential for JEV replication [49]. In 2023 the first commercial CRISPR knockout library was released by Cellecta, Inc. [144]. In a two-vector lentiviral CRISPR system, cells expressing the Cas9 protein are generated first, and then transduced with the gRNA library.

Recently developed base and prime editors offer more precise modifications without double-strand breaks and donor DNA templates, thereby reducing concerns about genomic instability and off-target effects. Base editing was first performed in pigs in 2018 to model ablepharon macrostomia syndrome and albinism caused by one base change. Porcine fibroblasts were transfected with plasmids carrying CRISPR/Cas system with 84% efficiency, and 25 piglets were obtained through SCNT [145]. This approach has been extended to other diseases such as Hutchinson–Gilford Progeria Syndrome. Multiple sites were successfully edited in embryos and somatic cells via delivery of an RNA-encoding cytosine base editor [146]. A base editor in the form of RNA was delivered to porcine zygotes and fibroblasts to introduce a nonsense mutation in a xenogeneic antigen-related gene. The efficiency of base editing was 18.1–72.4% depending on the number of gRNAs [147].

Prime editing was for the first time employed in pigs in 2023. Multiple modifications such as mutating of pegRNA, treatment with cephalosporin C zinc salt to improve reverse transcription, and using histone deacetylase inhibitors to increase prime editor expression, allowed an increase in prime editing of porcine embryonic fibroblasts up to 122-fold [148]. A novel 2025 study describes a new reverse transcriptase derived from a porcine endogenous retrovirus. The optimized prime editing system demonstrated 102-fold higher editing efficiency in porcine fibroblasts and allowed the modification of three genes to model Alzheimer’s disease. Edited cells were used to produce 29 live-born piglets from four sows via SCNT [149].

Each delivery platform has its strengths and limitations, summarized in Table 1. The choice depends on the required efficiency, precision, cost, timeline, biosafety, and experimental goals. Equipment availability, personnel expertise, and financial resources are also important factors limiting the use of the delivery methods. For traditional CRISPR/Cas induced knockout, RNP delivery is the best option for reduced mosaicism, faster action, and less risk. It is now a gold standard for genome editing in pigs. More complicated approaches, such as knock-in, base editing, and prime editing, usually have lower efficiency and may require different strategies. Thus, a comprehensive set of tools for all types of pig genome editing has been developed to create animals with improved agricultural traits.

## 5. Application of Genome Editing to Improve Agricultural Traits in Pig

Despite its novelty, genome editing has been used in pigs multiple times. Some genome-edited pig lines have reached or are close to commercial approval. In 2023, the FDA approved gene-edited pigs with male sterility produced by Washington State University via the knockout of *NANOS2* gene. Sterility allows for implantation of stem cells from another boar that can create sperm with desired traits [151]. In 2025, PRRS-resistant pigs developed by PIC (Pig Improvement Company) were approved for human consumption [152]. The animals were originally created during one of the first CRISPR/Cas experiments with pigs at the University of Missouri in 2014 and have mutations in exon 7 of *CD163* gene [135]. Therefore, it took more than 10 years to gain approval from FDA.

This section systematically summarizes successful and unsuccessful cases of genome editing in improving pig agricultural traits, including virus resistance, increased productivity, temperature sensitivity, and introduction of transgenes using genome editing techniques.

### 5.1. Virus Resistance

PRRS-resistant pigs are relatively easy to produce, as demonstrated by the various research groups around the world, including those from China and the UK. It has been shown that disruption of the Scavenger Receptor Cysteine-Rich Domain 5 (SRCR5) of porcine *CD163* gene located on exon 7 guarantees complete resistance to viral infection without any adverse effects in pigs [153,154,155]. However, the CRISPR/Cas9 knockout of the surface protein SIGLEC1 (CD169) earlier considered a primary viral receptor showed that it is unnecessary for infectivity [27].

Pigs resistant to other viral infections might be the next to reach the market. However, approaches to achieve such resistance were not as efficient as the knockout of *CD163* gene against PRRS. One successful example is editing of the 2nd exon of the *ANPEP* gene, resulting in a premature stop codon and complete loss of protein activity. Genome-edited pigs were completely resistant to TGEV infection, as evidenced by the absence of virus in the feces and the viral antigen in ileum. However, they retained susceptibility to PEDV and PDCoV, demonstrating differences in the entry mechanisms of coronaviruses [156,157]. Double CD163/ANPEP knockout pigs were resistant to both PRRSV and TGEV [158].

The *CMP-N-glycolylneuraminic acid hydroxylase* (*CMAH*) gene was knocked out via the CRISPR/Cas9 system to protect pigs from PEDV. Genome-edited piglets showed a delayed onset and milder symptoms of disease, but they were not completely resistant to the infection [159].

Pigs with edited *DNAJC14* gene were resistant to infection with two non-cp pestiviruses, CSFV and BVDV, due to the prevention of their replication [38,160]. Knockout of the *PCBP1* gene significantly reduced CSFV infection in pigs due to the activation of the type I interferon [37].

Cell cultures of *ANTXR1* knockout pigs were completely resistant to SVA infection; however, in-frame mutation resulted only in partial resistance. But, it appeared that both type of mutations in *ANTXR1* result in a GAPO syndrome characterized by growth retardation, alopecia, pseudoanodontia, optic atrophy, and early death [40].

Genome-wide CRISPR/Cas9 screens helped to identify new genes involved in coronavirus infections. Transmembrane protein 41B (TMEM41B) was identified as a host factor required for coronavirus replication in pigs. *TMEM41B* knockout cells were unable to form the double-membrane vesicles necessary for TGEV replication [161].

The solute carrier family 35 member A1 (SLC35A1) and the VPS35 endosomal protein sorting factor like (VPS35L) were identified as a host factors required for PDCoV infection. As the SLC35A1 molecule is involved in the sialic acid synthesis pathway, knockout cells had reduced content of cell surface sialic acid. However the double knockout of *SLC35A1* and *ANPEP* could not block PDCoV infection completely, viral adsorption in genome-edited cells decreased [162]. *C16orf62* gene knockout contributed to downregulation of *ANPEP* expression at the cell surface and a remarkable decrease in the binding and internalization of PDCoV into host cells. Despite playing an important role in protein recycling in the endosomal compartment, C16orf62 disruption could not ensure full virus resistance [163].

Signal peptide peptidase 3 (SPPL3) was identified as a key factor involved in particle entry of four genera of pig coronaviruses by genome-wide CRISPR screening, but its role in resistance was not validated [164].

CRISPR screening also revealed that the *endoplasmic reticulum membrane complex* 3 (*EMC3*) and *calreticulin* (*CALR*) genes are critical for JEV entry and replication. Before that, key host factors facilitating JEV infection in pigs were unknown. Knockout of the *EMC3* gene significantly inhibited the replication of virus in porcine cells, and knockout of the *CALR* gene resulted in a strong resistance phenotype [49]. The CRISPR-Cas9-mediated cytosine base editor was used for the screening of pivotal variants for the *CALR* gene function using more than 450 gRNAs. However, mutations the introns induced by base editing inhibited JEV replication, and full resistance was not achieved using this approach [165].

A genome-wide CRISPR/Cas9 knockout screen revealed that *transmembrane protein 239 (TMEM239)* reduced replication of genotype II ASFV in porcine cells by preventing virus entry into early endosomes [166]. Editing of the *RELA* gene with ZFN to generate either 2 or 3 amino acid substitutions found in warthogs was not effective in achieving resistance to ASFV. Pigs with three substitutions demonstrated a delayed onset of clinical signs and less viral DNA load; however, animals with two substitutions did not differ from control [167,168]. Due to the economic importance of the disease and lack of success in identifying the ASFV receptor, there were approaches to edit the genome of the virus itself [169,170].

Knockout of the *transmembrane serine protease 2* gene (*TMPRSS2*) increased pig resistance to influenza, represented by delayed viral replication, lower virus titers, and significantly fewer lung lesions in the lower respiratory tract [41,42,137]. *Serine/threonine kinase 11* (*STK11*) was suggested essential for Swine influenza virus attachment based on the genome-wide CRISPR screens. However, in vivo validation was performed in mice and not pigs [171].

*Nectin 1* and *Nectin 2* knockout cells, as well as the cells with double gene knockout, had significantly reduced PRV growth due to the blocked cell-to-cell spread; however, virus attachment or internalization to cells was not affected enough to ensure resistance [44].

There were several approaches not to knock out, but to knock in genes to obtain virus-resistant pigs. Insertions are usually made to the Rosa26 locus which serves as a “safe harbor” in many species including humans [172]. Insertion of the *Radical S-adenosyl methionine domain containing 2* (*RSAD2*) gene with a broad antiviral activity, also known as viperin, resulted in reduced CSFV and PRV infection in pig cells [173].

Antiviral miR30-based small hairpin RNAs (shRNAs) designed for CSFV RNA interference were inserted into the porcine Rosa26 locus. Genome-edited pigs demonstrated a significant reduction in virus replication. Clinical manifestations of the disease and viremia levels were significantly lower than in control animals [174].

Therefore, despite a large amount of genes associated with virus resistance, editing of only few of them gave the desired result. In total, only the *CD163*, *ANPEP*, *DNAJC14*, and *ANTXR1* targets proved efficient in vivo.

### 5.2. Increased Productivity

The *MSTN* gene is the most popular target for genome editing in various species. Since it has been discovered that natural mutation in the exon 3 causes leg weakness, many approaches to avoid this negative effect have been developed. Chinese-bred Meishan pigs were the first *MSTN* loss-of-function mutants created via ZFN technology. Targeting of exon 2 caused myofiber hyperplasia resulting in 100% more muscle and 12% greater lean meat yield than in control animals at 8 months, as well as normal fertility [175]. Deletions in exons 2 and 3 induced via TALEN contributed to a 170% increase in muscle mass in piglets, but most of them were stillborn, or died within 24 h after birth. The only surviving piglet had weak hindlimbs and was incapable of mating [176]. Heterozygous animals edited via TALEN targeting exon 1 retained reproduction ability, but litter size was significantly lower. Homozygous mutants were unhealthy, showing signs of dyspnoea, large tongues, and umbilical hernias [177]. Knockout mutant piglets created via CRISPR/Cas9 with two gRNAs targeting the 3d exon had 15% increases in birth weight, enlarged tongues, and intermuscular boundaries, and they all died within a week [178]. In general, targeting of exon 3 of *MSTN* gene replicating a naturally occurring 11 bp deletion found in cattle caused unwanted effects in pigs. Low survival rates in early studies may also be related to the use of SCNT before RNP complexes were introduced.

Fan et al. proved that unlike mutation in exon 3 of *MSTN* gene, deletion of 40 bp from exon 1 did not cause any signs of leg weakness due to the preservation of the key amino acid C339, which is required for MSTN dimerization. In 2022, the number of CRISPR/Cas9 genome-edited pigs with this beneficial mutation exceeded 1000 [179].

The knockout of *F-Box Protein 40* (*FBXO40*) gene in pigs via CRISPR/Cas9 caused slight muscle hypertrophy, resulting in 32.7% increase in cross-sectional area of myofibers and just 4% increase in muscle mass. There were no detectable pathological changes in major organs, as well as pH, drip loss, fat content, moisture, and protein content [180].

Editing the zinc finger BED-type containing 6 (ZBED6) repressor binding sites in the 3d intron of the *IGF-2* gene significantly increased IGF-2 expression and improved muscle development in Chinese Liang Guang Small Spotted pigs. The conserved GCTCG motif was removed by the CRISPR/Cas9 system to eliminate the inhibitory effect of ZBED6. The only one piglet with the biallelic mutation remained healthy and exhibited accelerated skeletal muscle growth, increasing body weight by 32% at the age of 338 days, compared to the control. Analysis of embryonic fibroblasts revealed increased cell proliferation, enhanced myogenesis, and reduced apoptosis [181].

The knock-in of the *fatty acid desaturase* (*Fat-1*) and *IGF-1* genes via CRISPR/Cas9 was performed to promote growth and resistance to cancer, as well as to cardiovascular and neurological diseases. Fat-1 can convert n-6 polyunsaturated fatty acid (PUFAs) into n-3 PUFAs. Genome-edited piglets exhibited increased IGF-1 expression in the gastrocnemius muscle and significantly reduced ratios of n-6 PUFAs/n-3 PUFAs. For example, linolenic acid was reduced by 1.76-fold; however, alpha-linolenic acid was increased by 2.44 times and docosahexaenoic acid by 2.09 times. The overall health condition of the animals was not evaluated [182].

Deletion of 43 bp in exon 2 of the *LGALS12* gene via CRISPR/Cas9 suppressed cell proliferation and promoted lipolysis in intramuscular and subcutaneous porcine adipocyte cell cultures. However, experiments were only carried out in cell cultures, and the obtained data suggest that knockout of the *LGALS12* gene in pigs might contribute to leaner meat and reduced fat accumulation [183].

Two promoter complexes controlling growth hormone, *Growth hormone-releasing peptide 1* (*GHR1A*) and *GHRP2*, were deleted via the CRISPR/Cas9 system. GHRP2-deficient pigs were superior in birth weight, weaning weight, and body weight at the age of 260 days. Pigs with the deletion of *GHRP1* did not differ from the control group [184].

### 5.3. Temperature Sensitivity

There was a successful approach to increase the amounts of brown adipose tissue in pigs by editing *UCP1* gene. Insertion of the mouse *UCP1* cDNA driven by the adipose tissue-specific adiponectin promoter into the porcine endogenous UCP1 locus improved the ability to maintain body temperature and caused a 4.89% decrease in fat deposition, 26% thinner back fat, and a 24% reduction in the ratios of fat content to the carcass fat percentage. Genome-edited piglets better maintained body temperature: even at 4 °C, their temperature remained above 38 °C, whereas control animals displayed a continued decrease in body temperature. This process was accompanied by increased lipolysis, as evidenced by an increase in plasma free fatty acid concentrations and a decrease in triglyceride levels [185].

### 5.4. Knock-In

CRISPR/Cas9-mediated integration of a large transgene cassette carrying b-glucanase, xylanase, and phytase genes into CEP112 locus allowed the production of pigs with significant enzyme activity that increased with age. Moreover, it has been suggested that CEP112 locus is more efficient for gene insertion than ROSA26 in pigs [186].

CRISPR/Cas9-induced knock-in of *peroxisome proliferator-activated receptor gamma* (*PPARγ*) gene improved oxidative fiber formation and content of intramuscular fat in pig skeletal muscle due to the enhanced adipocyte differentiation [187].

Knock-in of a *porcine β-defensin 2* (*PBD-2*) gene, encoding a peptide with antimicrobial, immunomodulatory, and growth-promoting activity, into the Rosa26 locus contributed to a significant increase in the antimicrobial properties of the cell cultures produced from transgenic pigs [188].

*BMPR-IB* gene was targeted in pigs to improve reproductive performance. However, the aim of the research was to generate the A746G mutation which was associated with increased ovulation rates and litter size in sheep; using CRISPR/Cas9 and donor DNA, BMPR-IB knockout piglets were obtained unexpectedly. All of them were unable to stand and walk normally and had skeletal dysplasia [57].

## 6. Remaining Challenges and Opportunities in Pig Genome Editing

The use of genome editing to identify viral receptors and create resistant pigs is advancing rapidly; however, editing of genes responsible for other traits is relatively rare. Despite the extensive data on the genetic basis for pig agricultural traits, only a few genes are used to accelerate the breeding process. Although convincing evidence of the involvement of many genes in the development of certain diseases and traits has been presented based on the processing of GWAS, transcriptome analysis, NGS sequencing data, and CRISPR screens, application of these results remains very limited. Many promising virus receptors and host factors identified via CRISPR screens, such as SPPL3, TMEM41B, and EMC3, remain validated only at the cellular level, and their role is still not confirmed in live animals (Table 2). There are several reasons for the limited application of genome editing in agriculture.

First, many agricultural traits are polygenic, and editing of one gene cannot fully solve the problem. But, generation of viable homozygus piglets with multiple edits is still very complicated. For example, however a single *MSTN* gene involved in lameness has been edited, it is not enough to combat this polygenic disease. Other genes associated with lameness remain neglected (Table 2). The problem of damaging behaviors has been studied to an even lesser extent [61].

Second, none of the agriculturally oriented CRISPR/Cas events currently involve effectors, base editors, or prime editors, which demonstrates the technology’s enormous untapped potential far beyond simple knockouts and knock-ins. Genome editing allows mimicking any natural or pre-designed mutation and regulating expression of any gene at any development stage. But application of these new approaches is delayed in pigs, because they are much more complex and expensive than CRISPR/Cas9 RNPs, and the required proteins are not commercially available.

Third, there are public concerns about genome editing, which often center on food safety and potential ecological risks. However, this technology can reduce pain inflicted on animals and improve their health and adaptability to the farm conditions. Edits that remove the need for other stressful situations, such as aggression from other pigs or vaccination, also have clear benefits for pig welfare. The safety of animals resistant to viruses, bacteria, and parasites for consumers is objectively more important than the possible off-target effects of editing, which can be minimized and monitored. Moreover, when a dangerous infection is detected, all animals on the farm are usually slaughtered, which is obviously inhumane.

Genome editing technology provides unique opportunities to verify the role of mutations associated with the trait of interest through big data analysis. Identification of important polymorphisms responsible for phenotypic effects might be tricky. The major candidate genes suggested in different studies rarely coincide and are often breed-specific. For example, 30 SNPs from 26 genes, primarily *MTHFR*, *WNT2*, *APOE*, *BMP8*, *GNRHR*, and *OXTR*, were associated with lameness and lower fat content in pigs [189]. Another study considered this disease as osteochondrosis leading to dysfunctions of bone and joints, caused by polymorphisms in *KRT8*, *FAF*, and *PTH1R* genes in Duroc × Pietrain populations [58]. However, *Matrix gla protein* (*MGP*) was proposed as a potential candidate gene for the development of osteochondrosis in pigs of the same breeds in another study [190]. Several studies discovered quantitative trait loci (QTL) for lameness: 42 on chromosomes four and seven for White Duroc × Erhualian intercross [191], and 11 on eight chromosomes for Duroc × Pietrain [192], but no significant QTL in Landrace pigs [193]. Despite long-term research into the problem, genetic predisposition tests for leg weakness have never been created. CRISPR/Cas technique could help to validate the role of these candidate genes and either direct the breeding programs or generate the disease-resistant pig.

Some novel ideas to improve pig farming have been proposed that were previously unimaginable and can only be implemented by CRISPR/Cas genome editing. The idea of compassion and respect for animals implies the rejection of such mutilating practices as castration and cutting off tails [194]. It seems possible to create tailless pigs, as well as shift the sex ratio in the litter towards females. Knockout of the sex-determining region on the Y chromosome (SRY) can potentially suppress testis development to obtain a female phenotype in pigs, just like in mice and rabbits [195].

Pigs are hosts to parasites dangerous to humans, such as the pork tapeworm *Taenia solium*. Despite the existence of vaccines, raw pork is still considered dangerous to consume [196]. Resistance to the parasite may have genetic nature. Pigs from three rural Mexican stud families demonstrated significant differences in the prevalence and intensity of infection from 4.4 to 25% [197]. Knock-in of genes encoding *T. solium* antigens (e.g., TSOL18, a proven vaccine target) and determining of resistance genes could contribute to production of safer pork meat.

Therefore, despite the challenges, it is undeniable that genome editing has the potential to revolutionize agriculture. Proper funding of agricultural research and collaboration between science and industry will facilitate the rapid adoption of genome editing technologies and improve pig productivity and welfare. Educating the public and harmonizing international regulations could streamline approval and implementation processes, helping to address the global food crisis.

## 7. Conclusions

Genetics is fundamental and irreplaceable in solving the major problems of pig productivity and pork quality in a sustainable and effective manner. Genome editing technology has made it possible to achieve traits such as virus resistance, which is impossible using traditional breeding methods. But, at the moment, application of pig genome editing in agriculture faces significant challenges, which range from regulatory hurdles to practical limitations in commercial applications, and requires global regulatory alignment. Scaling up the technology is currently difficult due to the requirement for specialized infrastructure, since pig herds cannot be maintained in laboratory conditions. Problems of embryo mosaicism, low efficiency of pig cloning, and the polygenic nature of important agricultural traits have not yet been fully resolved, although significant progress has been made in this regard. The SCNT allows us to obtain homozygous animals in a single generation, and much effort is being made to increase the efficiency of pig cloning. Certain edits, such as *MSTN* gene knockout, can also have physiological side effects. It remains to be determined which mutations allow the avoidance of undesirable consequences. CRISPR screens conducted in cell cultures can be employed for this purpose. This approach also helps identify potential target genes involved in various processes such as virus attachment and entry.

Embryo editing and SCNT technologies are developed in pigs better than in other animals, and in vivo editing with AAV vectors has recently come into use. RNP complexes delivery became the gold standard for CRISPR/Cas, ensuring that integration of foreign DNA will not occur.

Although porcine genomes are still mostly edited for biomedical purposes, and rarely for agriculture, certain achievements have been made. The *CD163*, *ANPEP*, *DNAJC14*, and *ANTXR1* knockout pigs proved resistant to viral infections. Genome-edited pigs with male sterility and PRRS-resistant pigs were recently approved for human consumption. Editing of the *MSTN*, *FBXO40*, and *ZBED6* genes can increase productivity of pork production. Knock-in of the functional *UCP1* gene can increase cold tolerance in piglets. Thus, genome editing has great potential not only against pig viral diseases but also in improving meat quality, stress tolerance, and studying polygenic agricultural traits. Genome editing has potential to overcome limitations of traditional breeding, such as slow genetic gains and restricted allele availability across breeds or species.

## Figures and Tables

**Table 1:** Delivery platforms for genome modification in pigs.

Platform	Typical Delivery Method	Achievable Transformation Efficiency	Key Advantages	Key Limitations	Purpose
Plasmid DNA	Transfection/microinjection of fibroblasts/zygotes	84% [145]	Inexpensive and simple. Supports expression of large constructs	Low efficiency, random integration	Transgenesis, transient expression and genome editing
Transposon Vector	Transfection/microinjection of a plasmid with target insertion with transposase as separate plasmid, mRNA or protein into cells/zygotes.	8% [126,127,128,129]	Highly efficient genomic integration. Extremely large cargos (>100 kb for piggyBac).	Random integration, risk of insertional mutagenesis	Efficient transgenesis, especially suitable for insertion of multiple genes (e.g., for xenotransplantation).
Recombinase Vector	Transfection of fibroblasts, injection of viral vector into the organ of a genetically engineered pig	81.9% [130]	Precise excision, inversion, or insertion. Can be delivered to specific tissues in vivo for conditional knockout studies	Requires a pre-existing genetically modified animal with recombination sites.	Targeted integration, tissue-specific or inducible gene activation or inactivation in a live animal.
LVV vector	In vitro viral transduction into cells/zygotes by incubation. Local injection.	70% [19]	Very high delivery efficiency and transgenic yield. Long-term, stable expression, especially in dividing cells. Ideal for stable modification of fibroblasts. Can target non-dividing cells	Complex production in packaging cell lines to supply missing viral proteins. Immune response concerns, poor embryo survival. Integration into the host genome. Limited cargo capacity (~8 kbp)	High-efficiency transgenesis. Generation of stable, Cas-expressing cell lines, which are later edited by sgRNA delivery
AAV vector	In vivo injection (Intravenous, in utero, oviductal, into specific organs), in vitro viral transduction by incubation	64% [150]	Non-integrating. Excellent for in vivo delivery to specific tissues, in vivo embryo editing (oviductal injection) and SMGT. Low immunogenicity.	Complex production in packaging cell lines to supply missing viral proteins. Limited cargo capacity (~4.7 kbp). Expression decreases with cell division	In vivo genome editing and gene therapy.
Sendai RNA virus	In vitro viral transduction of fibroblasts by Incubation	30% [123]	Non-integrating. Non-DNA. High transduction efficiency.	Require specific packaging cells designed to express the fusion protein	Generating iPSCs in pigs by cellular reprogramming without genomic integration of transgenes, vaccine platform for animal diseases
Linear DNA (PCR fragments, linearized plasmids)	Co-delivery with CRISPR system (e.g., via microinjection or electroporation).	6–54% [138]	Simplest form of HDR template. Reduces risk of random plasmid integration.	Very low HDR efficiency, degradation, unstable expression	Serves as a template for homology-directed repair (HDR) for CRISPR-induced knock-in.
RNP (Ribonucleoprotein)	Transfection/microinjection of fibroblasts/zygotes	77% [140]	Rapid action. Highest on-target efficiency, lowest off-target effects. Minimal mosaicism in embryos. Transient (no integration)	Short half-life. Requires co-delivery of a separate template for knock-in. Requires protein production and in vitro RNA synthesis.	Gene knockouts. The gold standard for direct embryo editing.
RNA (TALEN or Cas9 mRNA + sgRNA)		100% [136,137]	longer window of activity, no need for protein synthesis	Requires in vitro RNA synthesis	Gene knockouts.

**Table 2:** Application of genome editing to improve agricultural traits.

Trait	Porcine Gene	Tagreted by Genome Editing	Confirmed in Live Animals
Temperature sensitivity	*UCP1*	yes	yes
Temperature sensitivity	*HSP70*	no	no
Allergic reactions	*GGTA1*	yes	yes
Male sterility	*NANOS2*	yes	yes
Multiple pregnancy	*ESR*	no	no
Porcine Stress Syndrome	*RYR1*	no	no
Meat productivity	*IGF-2*	yes	yes
	*IGF-1*	yes	yes
	*FBXO40*	yes	yes
	*GHR1A*	yes	yes
	*GHRP2*	yes	yes
Meat productivity, lameness	*MSTN*	yes	yes
Meat quality	*LGALS12*	no	no
	*PPARγ*	yes	yes
	*Fat-1*	yes	yes
	*CAST*	no	no
	*PRKAG3*	no	no
	*TMCO1*	no	no
	*LRATD1*	no	no
	*CKB*	no	no
	*H-FABP*	no	no
Anal atresia	*GLI2*	no	no
Myopathy	*SPTBN4*	no	no
Congenital splay leg syndrom	*FBXO32*	no	no
	*P311*	no	no
	*HOMER1*	no	no
Antimicrobial qualities	*FUT1*	no	no
	*MUC4*	no	no
	*NRAMP1*	no	no
	*PBD-2*	yes	yes
PRRSV, TGEV, PEDV, PDCoV, CSFV, PRV resistance	*heparan sulfate*	no	no
CSFV, PRV resistance	*RSAD2*	yes	no
PRRSV resistance	*vimentin*	no	no
	*CD151*	no	no
	*RGS16*	no	no
	*GBP1*	no	no
	*GBP5*	no	no
PRRSV and PCV resistance	*CD163*	yes	yes
	*CD169*	no	no
	*CD209*	no	no
TGEV, PEDV, PDCoV resistance	*ANPEP*	yes	yes
	integrins *αvβ1, αvβ3*, *αvβ6*, *and αvβ8*	no	no
	*SPPL3*	yes	no
TGEV resistance	*TMEM41B*	yes	no
PDCoV resistance	*SLC35A1*	yes	no
	*VPS35L*	yes	no
	*C16orf62*	yes	no
JEV resistance	*EMC3*	yes	no
	*CALR*	yes	no
PEDV resistance	*CMAH*	yes	yes
CSFV resistance	*CD46*	no	no
	*PCBP1*	no	no
BVDV, CSFV resistance	*DNAJC14*	yes	yes
SVA resistance	*ANTXR1*	yes	yes
SVA, influenza resistance	*TMPRSS2*	yes	yes
CSFV and influenza A resistance	*Mx1*	no	no
Influenza resistance	*STK11*	no	no
PRV resistance	*Nectin 1 and 2*	yes	no
ASFV resistance	*RELA*	yes	yes
	*CD1d*	yes	yes
	*TMEM239*	yes	no
Lameness	*CNR2*	no	no
	*Lin28a*	no	no
	*ZBTB40*	no	no
	*MMP3*	no	no
	*TGFβ1*	no	no
	*BMPR-IB*	yes	yes
	*FAF1*	no	no
	*PTH1R*	no	no
	*KRT8*	no	no
Damaging behaviors	*DRD2*	no	no
	*SLC6A4*	no	no
	*MAOA*	no	no
	*RYR1*	no	no
Boar taint	*GnRF*	no	no
	*cytochrome P450*	no	no
	*SULT2A1 and SULT2B1*	no	no
	*LHB*	no	no
	*3βHSD*	no	no
	*17βHSD*	no	no

## Data Availability

No new data were created or analyzed in this study. Data sharing is not applicable to this article.
